# Predicting residue‐specific qualities of individual protein models using residual neural networks and graph neural networks

**DOI:** 10.1002/prot.26400

**Published:** 2022-07-30

**Authors:** Chenguang Zhao, Tong Liu, Zheng Wang

**Affiliations:** ^1^ Department of Computer Science University of Miami Coral Gables Florida USA

**Keywords:** CASP14 QA assessment, estimation of protein model accuracy, graph neural network, protein model quality assessment, protein structure prediction, quality assessment for AlphaFold2 models, residual neural networks

## Abstract

The estimation of protein model accuracy (EMA) or model quality assessment (QA) is important for protein structure prediction. An accurate EMA algorithm can guide the refinement of models or pick the best model or best parts of models from a pool of predicted tertiary structures. We developed two novel methods: MASS2 and LAW, for predicting residue‐specific or local qualities of individual models, which incorporate residual neural networks and graph neural networks, respectively. These two methods use similar features extracted from protein models but different architectures of neural networks to predict the local accuracies of single models. MASS2 and LAW participated in the QA category of CASP14, and according to our evaluations based on CASP14 official criteria, MASS2 and LAW are the best and second‐best methods based on the *Z*‐scores of ASE/100, AUC, and ULR‐1.*F*1. We also evaluated MASS2, LAW, and the residue‐specific predicted deviations (between model and native structure) generated by AlphaFold2 on CASP14 AlphaFold2 tertiary structure (TS) models. LAW achieved comparable or better performances compared to the predicted deviations generated by AlphaFold2 on AlphaFold2 TS models, even though LAW was not trained on any AlphaFold2 TS models. Specifically, LAW performed better on AUC and ULR scores, and AlphaFold2 performed better on ASE scores. This means that AlphaFold2 is better at predicting deviations, but LAW is better at classifying accurate and inaccurate residues and detecting unreliable local regions. MASS2 and LAW can be freely accessed from http://dna.cs.miami.edu/MASS2-CASP14/ and http://dna.cs.miami.edu/LAW-CASP14/, respectively.

## 1 INTRODUCTION

Protein tertiary structure (TS) predictors generate a large number of conformational decoys, which are predicted 3D structures, on the same target residue sequence, and the global and local accuracies of the decoys vary.^1^ Estimation of model accuracy (EMA) or quality assessment (QA) plays an essential role in selecting the best models from a pool of decoys. Local quality scores, such as deviations in angstroms, *S‐*score, or LDDT,^2^ are usually used to describe the deviation for residues and guide the refinement of the model, whereas the global quality scores that indicate the quality of the entire model, such as the global distance test_total score (GDT_TS),^3^ are used to distinguish the best model.

As AlphaFold2^4^ achieved great success in TS predictions, the community of CASP14 has started to discuss the future role of QA.^5^ Although the TS models generated by AlphaFold2 have achieved promising accuracies, the benchmark of the confidence measures or residue‐specific deviations generated by AlphaFold2 and the performance comparisons of which with other QA methods were not performed in CASP14. This was because AlphaFold2 was registered as a human TS predictor in CASP14, and only the TS models from server predictors were sent to CASP14 QA methods for quality‐assessment predictions. To fill up the gap, in this work, we benchmarked the performance of the confidence measures generated by AlphaFold2 and compared it with our QA methods.

Most of the recent single‐model EMA methods use machine learning algorithms. DeepAccNet^6^ applies 2D convolution to the global environment and 3D convolution to the atomic environment to estimate the distance error. QDeep^7^ combines residue‐level ensemble classifications into local accuracy scores. Specifically, QDeep trains multiple residual neural networks (ResNets) based on different distance‐error thresholds. DeepQA^8^ applies a deep belief network on structural and physicochemical characteristics to estimate the quality of models. SMOQ^9^ is an SVM‐based method using secondary structures (SS), solvent accessibilities, and residue–residue contacts to predict the per‐residue distance deviation with a single‐model input. In our previous study,^10^ we designed four single‐model methods based on stacked denoising autoencoders, SVM, or the combination of them, which have achieved relatively better performance for the residues that have the native deviations greater than $5\;\overset{{}^{\circ}}{A}$. ProQ3D^11^ improved the Pearson correlation coefficient of local model quality from 0.72 as in ProQ2^12^ to 0.77 benchmarked on the CASP11 dataset by changing the SVM to multi‐layer perceptrons. The authors of QAcon^13^ found that the performance of its two‐layer neural network was improved by adding the novel feature of residue‐residue contact information, which illustrates the value of using the contact information in protein QA. Our previously developed MASS^14^ is a single‐model method that only predicts global quality scores using random forests based on six protein statistical potentials that indicate structural or energetic properties of protein models. In comparison, one of the methods presented in this article, MASS2, was trained to predict residue‐specific qualities and was based on completely different methodologies.

In CASP14, a few methods used graph neural network (GNN) into EMA for the first time. GraphQA^15^ represents a protein molecule as a graph by constructing edges between either spatially close or sequentially consecutive residues. GraphQA encodes spatial and sequential distance as edge features and adopts amino acid sequences, multiple sequence alignment (MSA), dihedral angles, and SS as node features. And then, GraphQA applies a GCN^16^ to predict local and global quality scores. GraphQA co‐optimizes two local and five global quality scores with weighted summed mean squared losses. However, the co‐optimization of GraphQA reduces the performance of local scores.^15^ VoroCNN^17^ constructs a graph from the model using a Voronoi diagram tool^18^ and predicts local scores by employing a graph convolutional neural network (GCNs). ProteinGCN^19^ generates features from structural and inter‐atomic orientations and distances and applies another type of GCN^20^ to the graph of *k*‐nearest residues.

The methods discussed in this paper, named MASS2 and LAW (LAW is an abbreviation of “local assessment of protein models using graph networks”), also participated in CASP14, which used ResNet and GNN, respectively. These two methods performed better than the other CASP14 methods using similar methods according to our implementation of the CASP official evaluation criteria.

## 2 METHODS AND MATERIALS

### 2.1 Overview

**FIGURE 1:**
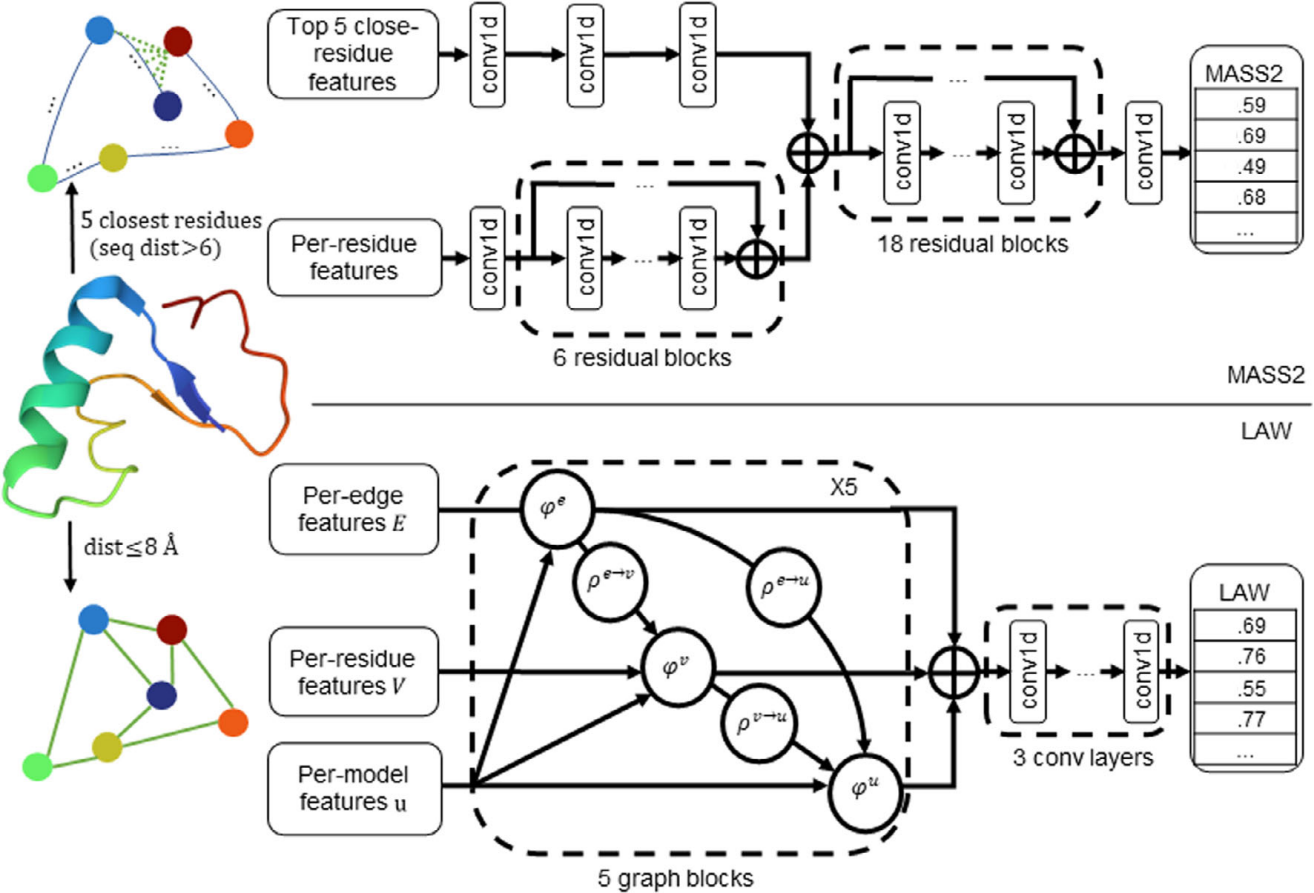
Deep learning architectures of MASS2 and LAW. MASS2 applies residual networks to the features of individual residues and their five closest residues. LAW represents the model as a graph through proximity residues and applies a graph neural network to the graph.

Figure 1 shows the architectures of deep learning networks for predicting the local quality of a model. The model and residues are rainbow‐colored, and the colors are from dark blue (the first residue) to dark red (the last residue). MASS2 and LAW are implemented with Resnet and GNN, respectively. These two methods use similar features but different neural networks. The MASS2 utilizes the 3D closeness information by combining the features of the spatially close but sequentially far residues from each residue. However, the LAW captures the 3D structure of a predicted model by creating a graph for nearby residues with a distance cutoff to the target residue. Both methods predict per‐residue local quality scores.

We used the models from CASP7 to CASP12 for training and validation, where models are trimmed into evaluation units (EUs).^21^ For each residue of a protein model, we generated features of six categories: one‐hot encoding of amino acid sequence (1 × 21), position‐specific scoring matrix (PSSM) created by PSI‐BLAST with the MSA (1 × 20), normalized Rosetta energies (1 × 20), SOV_refine^22^ local scores (1 × 6) and global scores (1 × 6) between predicted (from sequences) and parsed (from models) SS and solvent accessibilities, MASS protein statistical potentials^14^ (1 × 6), and (6) sinusoidal positional encoding (1 × 4).

### 2.2 The architecture of MASS2

The deep learning network architecture of MASS2 contains two branches. The input of the first branch is the concatenated features of the five spatially closest residues (sequential distances >6) for each target residue. The features pass through three convolutional layers. In contrast, the input of the second branch is the per‐residue features of a model, where the second branch contains a convolutional layer and six residual blocks.

The concatenated outputs of two branches are fed into another 18 residual blocks and a fully connected layer, which reduces the dimension of features to one. The output after the sigmoid function is the predicted *S*‐score that is further converted to residue‐specific deviation as follows: $\text{deviation}=d_{0}\sqrt{1/S-\text{score}-1}$, where $d_{0}$ is a constant set to 3. The architecture of MASS2 has a total of 24 residual blocks. Each block has two Conv1D‐BatchNorm‐Dropout‐LeakyReLU layers. At the end of a block, the input of the block and the output of LeakyReLU are combined and outputted.

The features of residues for MASS2 contain one‐hot encoding, PSSM, normalized Rosetta energies, SOV_refine local and global scores, MASS protein statistical potentials, and sinusoidal positional encoding. MASS2 uses the duplicated SOV_refine global scores at each residue.

### 2.3 The architecture of LAW

The architecture of the GNN network used in LAW includes five graph blocks. A graph is generated for each model by adding edges to any two residues with a 3D Euclidean distance $\leq 8\;\overset{{}^{\circ}}{A}$. The edges are unweighted and bi‐directed, so each node is a sender and a receiver.

LAW contains five graph blocks and three convolutional layers. The inputs of the graph network are nodes E, edges V, and global features u. In the GNN framework,^16^ each graph block is a computation unit, which has “update” functions φ and “aggregation” functions ρ. The “update” functions are implemented with fully connected layers and a ReLU function, and the “aggregation” functions are implemented with scatter mean functions.^23^ The details in a computation unit are described below in three steps:

The first step is to update the edge features. We concatenate sender features, receiver features, edge features, and global features. Then we apply a Linear–ReLU–Linear layer to the concatenated features for updating edge features, in which the linear part is implemented as a fully connected layer in Pytorch.^23^

The second step is to update the node features. We pass the concatenated sender features and edge features into a Linear–ReLU–Linear layer. After that, we use the “aggregation” function $\rho^{e\to v}$ to average the edge features over receivers. The message from the edge features is passed to node features. And then, the concatenated node, edge, and global features are passed into another Linear–ReLU–Linear layer.

The third step is to update the global features. Before that, we use $\rho^{e\to u}$ to average edge features over receivers and then over graphs, and then we use $\rho^{v\to u}$ to average node feature over graphs. We concatenate the aggregated edge and node features with global features and update the global features.

We let the input features go through five graph blocks. After that, we averaged the edge features to receivers and concatenated the aggregated edge features with node features and global features. Then we applied three conventional layers to reduce the dimension of the node feature to one.

The node features for LAW contain one‐hot encoding, PSSM, normalized Rosetta energies, SOV_refine local scores, MASS protein statistical potentials, and sinusoidal positional encoding. The edge features contain Euclidean distances divided by eight, the cosine of two vectors from the origin of the axes to residues, and the sequential distance between two corresponding residues. The global features are the SOV_refine global scores for SS and solvent accessibility.

### 2.4 Features of MASS2 and LAW

#### 2.4.1 One‐hot encoding of amino acid sequence

We assume 20 unique amino acids are 20 binary variables, respectively. All other amino acid types that do not belong to these 20 amino acids are encoded to the 21st binary variable. Each amino acid is encoded to a vector of 21 variables where 1 is assigned to the corresponding amino acid type, and the 0s are assigned to all other amino acid types. A target sequence with length *L* is coded to a matrix of *L* × 21 with the one‐hot‐encoding method.

#### 2.4.2 Rosetta energy functions

Rosetta Energy Function 2015 (REF15) is a mathematical model parametrized for estimating energy related to biomolecule structure.^24^ Each residue receives 19 energy scores from REF15, and we use the sum up of 19 energy scores as the 20th energy score.

#### 2.4.3 Position‐specific scoring matrix

PSSM can capture evolutionary information by examining conserved positions from MSAs. Each amino acid of a target receives 20 position scores. The highly conserved positions have high scores, and the weakly conserved positions have low scores. The PSSM is generated using PSI‐BLAST.^25^

#### 2.4.4 SOV_refine scores

SOV_refine scores^22^ indicate the difference between sequence‐based and model‐based predictions of SS and solvent accessibility. We use DSSP^26^ to assign the SS and the relative solvent accessibilities (RSA) of a model, and we used SCRATCH^27^ to predict the SS and RSA from a sequence. The Q3, SOV'99,^28^ and SOV_refine^22^ scores are calculated between the assigned and predicted SSs and solvent accessibilities.

For each residue, the SOV_refine local scores are calculated by taking six SS or RSA scores from left and right and comparing sequence‐based and model‐based predictions using the tool SOV_refine.^22^ For each model, the SOV_refine global scores are calculated by comparing sequence‐based and model‐based all SS or RSA predictions using the tool SOV_refine.

#### 2.4.5 MASS protein statistical potentials

Our previous MASS system developed the MASS potentials^14^ as machine learning features. MASS potentials introduce energy functions and statistical potentials, capturing features from models. The potentials include pseudo‐bond angle potential, the accessible surface potential at the atomic level, sequence separation‐dependent potential, contact‐dependent potential, relative solvent accessibility potential, and volume‐dependent potential.

#### 2.4.6 Positional encoding

We use four sinusoidal positional encodings as the per‐residue features to make the deep learning network know the sequence order. For a position number pos, that ranges from 1 to *L*, the following values are used as the positional features: $\sin\left(\mathrm{pos}\right)$, $\;\cos\left(\mathrm{pos}\right)$, $\;\sin\left(\mathrm{pos}/100\right)$, and $\cos\left(\mathrm{pos}/100\right)$. This method assigns a unique encoding for each residue, allowing the deep learning network to recognize the positions of amino acids. Compared to the learned positional embeddings, the sinusoidal positional encoding is not limited to the length of input sequences. The sinusoidal method shows competitive performance to the learned positional encoding.^29^

### 2.5 Dataset

All training, validation, and benchmarking data were downloaded from http://predictioncenter.org/download_area/CASP14/. We trained MASS2 and LAW with the TS models from CASP7‐11 and validated them with models from CASP12. The models with GDT_TS ≤40 were filtered out. Table S1 shows the number of targets, EUs, and models for the training, validation, and benchmarks. The training and validation models are pruned to the EUs due to the availability of actual local quality scores.

Since both MASS2 and LAW participated in CASP14, the TS models of CASP14 were used as benchmarking datasets while the predictions made by MASS2 and LAW during CASP14 were evaluated based on the EU definition provided by CASP14. Details about evaluations will be discussed later.

### 2.6 AlphaFold2 TS models and predicted deviations

We downloaded the TS models and CASP14 LGA results of AlphaFold2 (group number: TS427) from https://predictioncenter.org/download_area/CASP14/. Since CASP14 required TS groups to provide error estimations in Angstroms in the temperature factor field, we treated the values reported in the temperature factor field of the TS models of AlphaFold2 as the residue‐specific predicted deviations.

### 2.7 Webservers

MASS2 and LAW can be freely accessed from http://dna.cs.miami.edu/MASS2-CASP14/ and http://dna.cs.miami.edu/LAW-CASP14/, respectively. The local QA of each model takes about 5 min. We retrained and updated the neural networks of MASS2 and LAW after target T1043 during the CASP14. The web servers are using the updated MASS2 and LAW.

## 3 EVALUATION

### 3.1 Global GDT_TS scores and native deviation

To get the global GDT_TS scores and native deviations, CASP14 performed a superposition of each TS model and the experimentally determined structure using LGA.^3^ The coordinate of carbon alpha was used to represent the location of a residue. In this article, we divided TS models into three categories according to global GDT_TS scores. Those with no more than 40 GDT_TS are bad models, those with no less than 60 GDT_TS are good models, and others are average models. We evaluated the performance of all CASP14 single‐model methods on three sets of TS models: the models with GDT_TS > 40, GDT_TS between 40 and 60, and any GDT_TS. The first set was also what CASP14 organizers officially used to evaluate QA predictors, which filtered out bad models. The second set allows us to explicitly check the performance of methods on the average models. The third setting is to examine the performance of methods on all models. The second and third sets were not used in CASP official evaluations.

We evaluated the model by converting local distances into *S*‐score, which was calculated as follows: $1/\left(1+{\left(\text{distance}/d_{0}\right)}^{2}\right)$, where distance is the native deviation in $\;\overset{{}^{\circ}}{A}$, and $d_{0}=5\;\overset{{}^{\circ}}{A}$. All CASP14 QA groups submitted predicted local or residue‐specific deviations in angstroms except the groups QA074, QA066, and QA280, which submitted scores between 0 and 1. We treated these submitted scores from these three QA predictors as *S*‐scores during our evaluations.

### 3.2 Averaged residue‐wise *S*‐score error

The averaged residue‐wise *S*‐score error (ASE) is defined as: $\mathrm{ASE}=\left(1-\frac{1}{N}\sum_{i=1}^{N}\mathrm{abs}\left(S\left(e_{i}\right)-S\left(d_{i}\right)\right)\right)\times 100$, where $S\left(e_{i}\right)$, is the *S*‐score of the estimated distance between the model and the native structure, and $S\left(d_{i}\right)$ is the *S*‐score of the distance between the model and the native structure. The final ASE scores are averaged over all models and then all EUs.

### 3.3 Area under the curve

After the superposition of the predicted and native structures, each residue in the predicted structure is classified into two classes: accurately modeled or inaccurately modeled. The residues are considered accurately modeled if the deviations to the native counterparts are less than $3.8\;\overset{{}^{\circ}}{A}$. By comparing the classes of residues generated from the predicted local quality scores and the classes of residues based on the superposition, we calculated the area under the curve (AUC). The final AUC scores were averaged over all models and then all EUs.

### 3.4 ULR‐1 and ULR‐2

Unlike the AUC, the unreliable local region (ULR) is designed to detect and evaluate continuous inaccurately modeled residues. If the deviation of a residue is less than $3.8\;\overset{{}^{\circ}}{A}$, the residue is accurately modeled in the TS model. A ULR is defined as a sequence of residues with at least three continuously inaccurately modeled residues. We then used two different ways of merging smaller ULRs into longer ULRs.

We defined the first way of merging, named ULR‐1, in the same way as in the official CASP14 evaluations (see “2.3 Methods for assessing local structure accuracy estimation” on page 3 of Ref 5), that was, if only one accurately modeled residue existed between two ULRs, then these two ULRs, together with the one residue in between, were merged into a longer ULR.

We defined the second way of merging, named ULR‐2, which was less stringent than ULR‐1, that was, if only one accurately modeled residue existed between one ULR and another inaccurately modeled residue or URL, then these ULRs or residues are merged into a longer ULR.

We compared the ULRs derived from the predicted local quality scores (referred to as predicted ULRs) and the ULRs parsed from the native deviations (referred to as true ULRs). If the start and end positions of a predicted ULR are within two residues of the start and end positions of the true ULR, then it is considered a correctly predicted URL. This is also what CASP14 organizers have done in their official evaluation (see “2.3 Methods for assessing local structure accuracy estimation” on page 3 of Ref 5).

The *F*1 score of ULRs was calculated as follows: $F1\;\text{score}=\left(2\times \text{precision}\times \text{recall}\right)/\left(\text{precision}+\text{recall}\right)$, and the final ULR.*F*1 scores were averaged over all models and then all EUs, which is in the same way as what is described in CASP14 official evaluations.^5^

**FIGURE 2:**
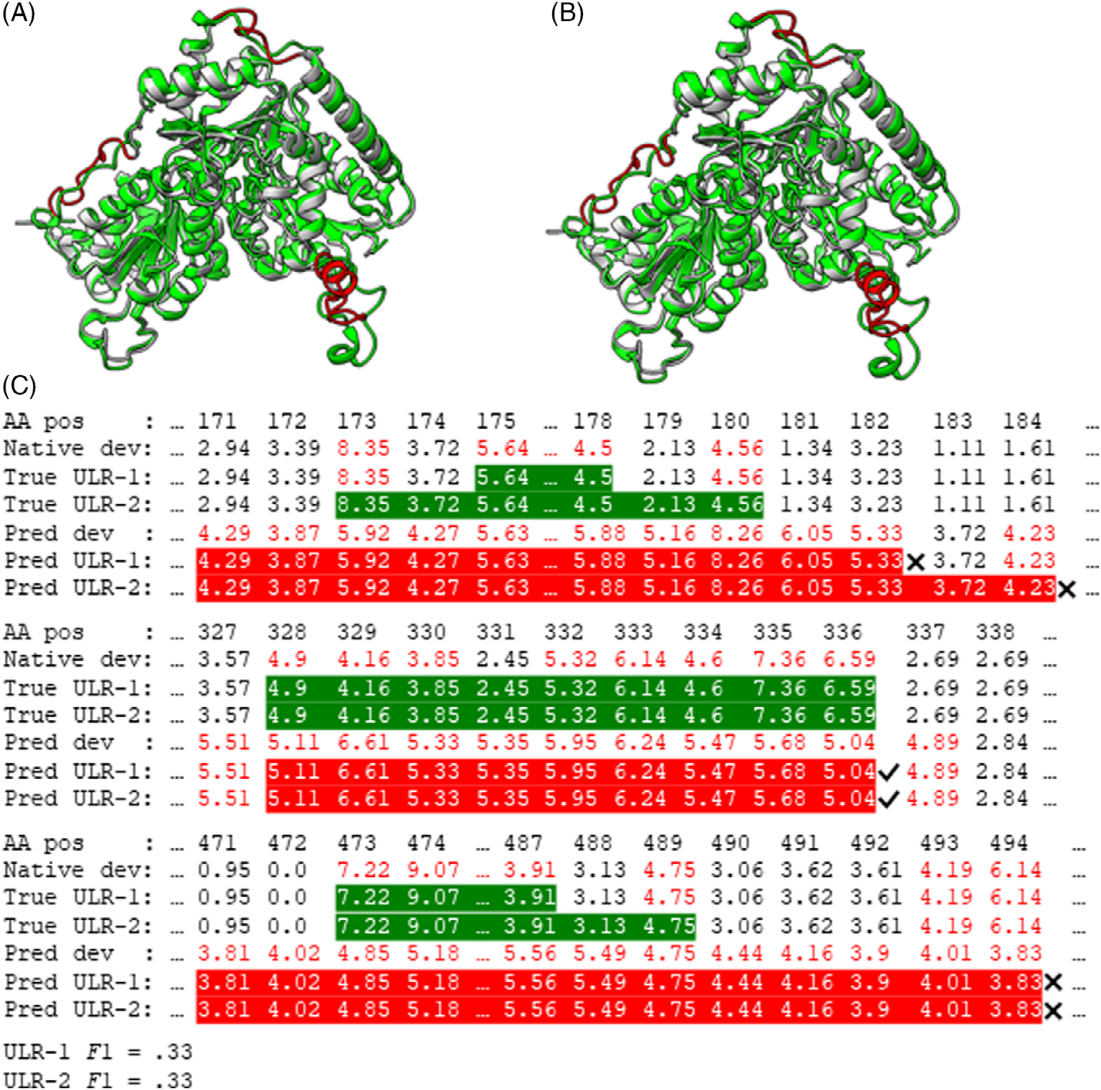
The ULR evaluation example on the same target and based on the same QA predictor (QA209) as in Figure 5A of CASP14 official QA evaluation paper.^5^ (A and B) Highlights the ULRs defined by two ways, ULR‐1 and ULR‐2, respectively, on the model T1076TS198_3‐D1 superposed with the native structure of target T1076. The red‐highlighted regions in (A) represents the ULRs defined in the same way as in CASP14 official QA evaluations, which is called ULR‐1 in our paper. The red‐highlighted regions in (B) represents the URLs defined by the second way of merging neighboring ULRs, named ULR‐2 in this paper. (C) Shows the amino acid position (AA pos), native deviation (Native dev), True ULR‐1, True ULR‐2, predicted deviation (Pred dev), predicted ULR‐1 (Pred ULR‐1), and predicted ULR‐2 (Pred ULR‐2). All deviations greater than $3.8\;\overset{{}^{\circ}}{A}$ are highlighted in red. In the lines of true ULR‐1 and true ULR‐2, the native deviation that belongs to true ULRs is highlighted with green background. In the lines of predicted ULR‐1 and predicted ULR‐2, the predicted deviations belonging to ULRs are highlighted with red background. Right (✔) and wrong (✖) symbols are used to indicate the correctness of predicted ULRs. The ULR‐1.*F*1 and ULR‐2.*F*1 are calculated at the bottom of the figure

Figure 2 shows the example of ULRs on a CASP14 target for a QA predictor. This example is also shown in Figure 5A of the CASP14 official evaluation paper.^5^ Figure 2A,B show the predicted ULR‐1 and ULR‐2 (red), respectively, and the details of defining ULRs are shown in Figure 2C. According to our observation and based on the data shown in Figure 2, the ULR‐1.*F*1 score for this example should be 0.33 ($\left[2\times 0.33\times 0.33\right)/\left(0.33+0.33\right)$) instead of 1.^5^ This may explain the differences in the evaluation results that we and CASP14 official evaluators generated.

Based on our observation and the data shown in Figure 2, we believed that our evaluations accurately followed the descriptions of how ULRs were defined and evaluated in the CASP14 official evaluation paper.^5^ To further prove that we strictly and accurately followed the same way as described by CASP14 official evaluators, we listed the GDT_TS, ASE, AUC, URL‐1.*F*1, ULR‐2.*F*1, SOV'99, and SOV_refine scores of all EUs for all CASP14 single‐model QA predictors in Supplementary File 1.

### 3.5 SOV_refine

As discussed in Section 2.4.4, SOV_refine scores can measure the similarity of two strings. Here, we used it to measure the predicted and true classes of correctly modeled or incorrectly modeled for all of the residues. We used 0 to represent an accurately modeled residue and 1 for inaccurately modeled residues. In this way, we generated a string of 0s and 1s from the predicted residue‐specific deviations of a QA group and another string from the true deviations. The similarity of these two strings was measured by the SOV_refine score. This is an evaluation criterion that was not used by the CASP14 official evaluations.

### 3.6 Evaluation details

To evaluate and compare the performances of MASS2 and LAW with other CASP14 predictors, we downloaded local quality scores generated by all of the single‐model CASP14 groups including our MASS2 and LAW. We also downloaded LGA results between TS models and native structures from CASP14 (https://predictioncenter.org/download_area/CASP14/). We evaluated the local quality estimations of all single‐model methods according to the steps discussed in the CASP14 official paper.^5^ For each evaluation criterion, we showed the detailed evaluation results of the top 10 CASP14 predictors based on our evaluations. Besides the top 10 predictors, Bhattacharya‐QDeep and GraphQA are two groups using similar methodologies (residual neural networks and GNNs) as MASS2 and LAW, respectively, and are also included to compare MASS2 and LAW with other methods that use similar methodologies.

## 4 RESULTS

**FIGURE 3:**
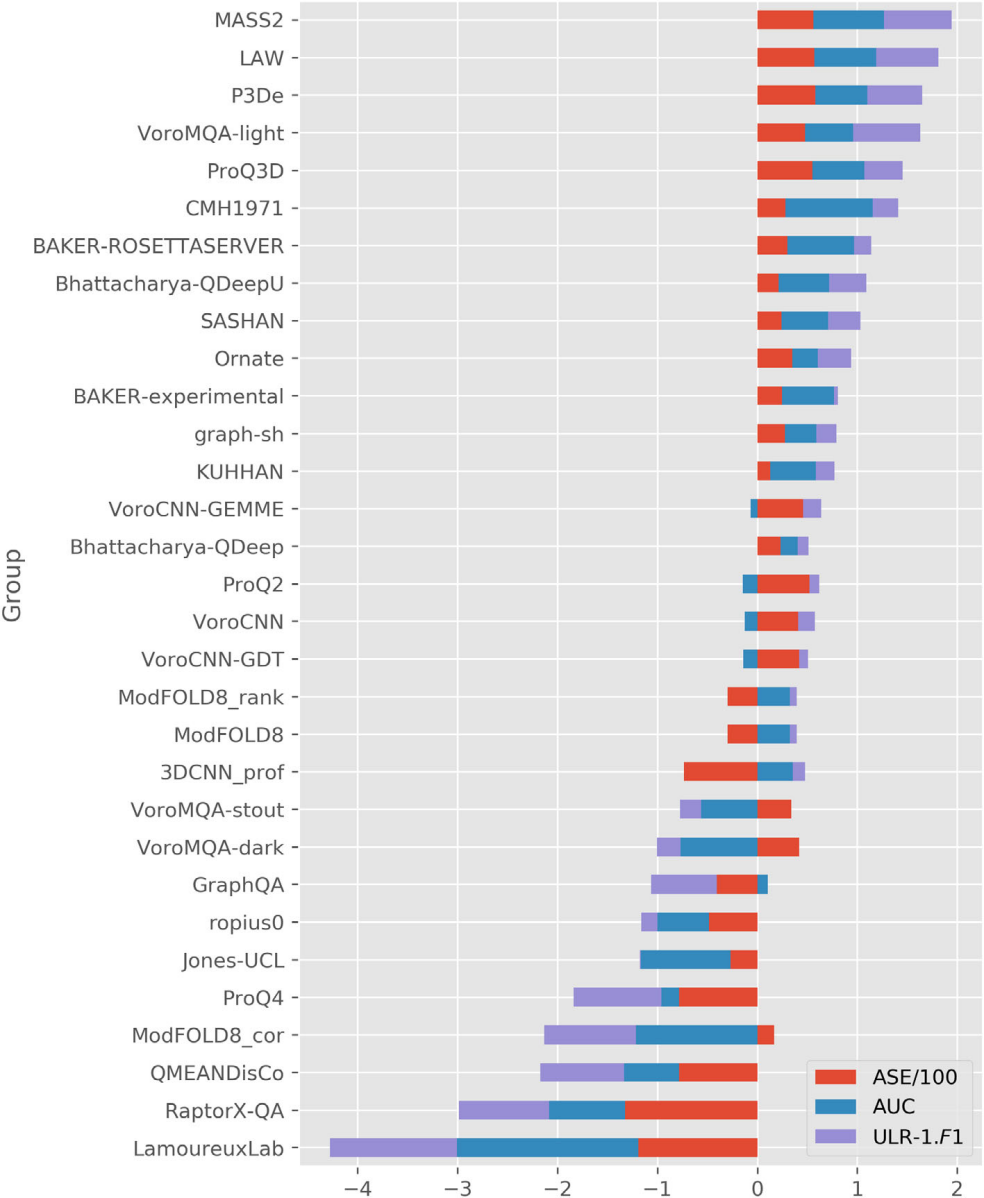
CASP14 groups ranked by the *Z*‐1 scores. *Z*‐1 score is the sum of averaged *Z* scores of ASE/100, AUC, and ULR‐1.*F*1 over all EUs

Figure 3 shows the ranking of 31 CASP14 single‐model groups according to *Z*‐1 scores. *Z*‐1 scores are the sum of averaged *Z* scores of ASE/100, AUC, and ULR‐1.*F*1 over all EUs. This is the same way of calculating *Z*‐1 scores as the CASP14 official paper.^5^ Based on our evaluation, MASS2 and LAW are the first and second methods in CASP14. Table 1 shows the details of the top 10 ranked groups plus Bhattacharya‐QDeep and GraphQA based on *Z*‐1 scores. Figure S1 shows the ranking of 31 CASP14 groups according to *Z*‐2 scores. *Z*‐2 scores are the sum of averaged *Z*‐scores of ASE/100, AUC, ULR‐2.*F*1, and SOV_refine over all EUs. Notice that this *Z*‐2 score was an evaluation criterion that CASP14 official evaluations did not use as it used ULR‐2 instead of ULR‐1. MASS2 and LAW are the first and third methods based on this criterion. Table S2 shows the details of *Z*‐2 scores of the top 10 ranked groups plus Bhattacharya‐QDeep and GraphQA.

**TABLE 1:** Selected CASP14 groups ranked by the *Z*‐1 scores

Ranked by *Z*‐1 scores	Groups	*Z*‐1 scores
1	MASS2	1.947
2	LAW	1.815
3	P3De	1.65
4	VoroMQA‐light	1.631
5	ProQ3D	1.454
6	CMH1971	1.411
7	BAKER‐ROSETTASERVER	1.142
8	Bhattacharya‐QDeepU	1.094
9	SASHAN	1.031
10	Ornate	0.942
15	Bhattacharya‐QDeep	0.514
23	GraphQA	−0.953

*Note*: *Z*‐1 scores are the sum of averaged *Z*‐scores of ASE/100, AUC, and ULR‐1 overall EUs.

Table 2 shows the ASE, AUC, ULR‐1.*F*1, ULR‐2.*F*1, SOV'99, and SOV_refine of MASS2, LAW, and the deviations generated by AlphaFold2 on the AlphaFold2 TS models in CASP14. Since AlphaFold2 usually generates high‐quality models, in addition to GDT_TS score thresholds >40 (40, 60), and (0, 100), we also benchmarked the performances of the three methods on the AlphaFold2 TS models having >90 GDT_TS scores. LAW performed well on AUC and ULR scores, and AlphaFold2 performed well on ASE scores. This means AlphaFold2 is better at predicting deviations, but LAW is better at classifying accurate and inaccurate residues and detecting ULRs. MASS2 performed well on ASE and AUC scores when the GDT_TS scores of models were between 40 and 60.

**TABLE 2:** Benchmarking results of MASS2, LAW, and the residue‐specific predicted deviations of AlphaFold2 (AF) on CASP14 AlphaFold2 TS models

GDT_TS	Groups	ASE	AUC	ULR‐1.*F*1	ULR‐2.*F*1	SOV'99	SOV_refine
>40	MASS2	74.54	0.61	0.068	0.073	0.286	0.199
	LAW	77.352	**0.662**	**0.083**	**0.104**	0.374	0.277
	AF	**82.984**	0.531	0.049	0.054	**0.5**	**0.385**
(40, 60)	MASS2	**65.22**	**0.559**	0.08	0.081	0.237	0.172
	LAW	64.341	0.542	**0.111**	**0.113**	0.233	0.176
	AF	62.237	0.548	0.017	0.02	**0.282**	**0.191**
>90	MASS2	84.32	0.494	‐	‐	0.489	0.318
	LAW	90.265	**0.522**	‐	‐	**0.75**	**0.616**
	AF	**94.662**	**0.522**	‐	‐	0.68	0.582
(0,100)	MASS2	74.517	0.609	0.067	0.072	0.285	0.198
	LAW	77.361	**0.663**	**0.081**	**0.102**	0.374	0.278
	AF	**82.938**	0.531	0.049	0.054	**0.5**	**0.385**

*Note*: Bold indicates the best scores among the three methods in each category. When the GDT_TS threshold is >90, there are few or no ULRs available. Therefore, all methods were not evaluated on ULRs as indicated by “‐.”

**FIGURE 4:**
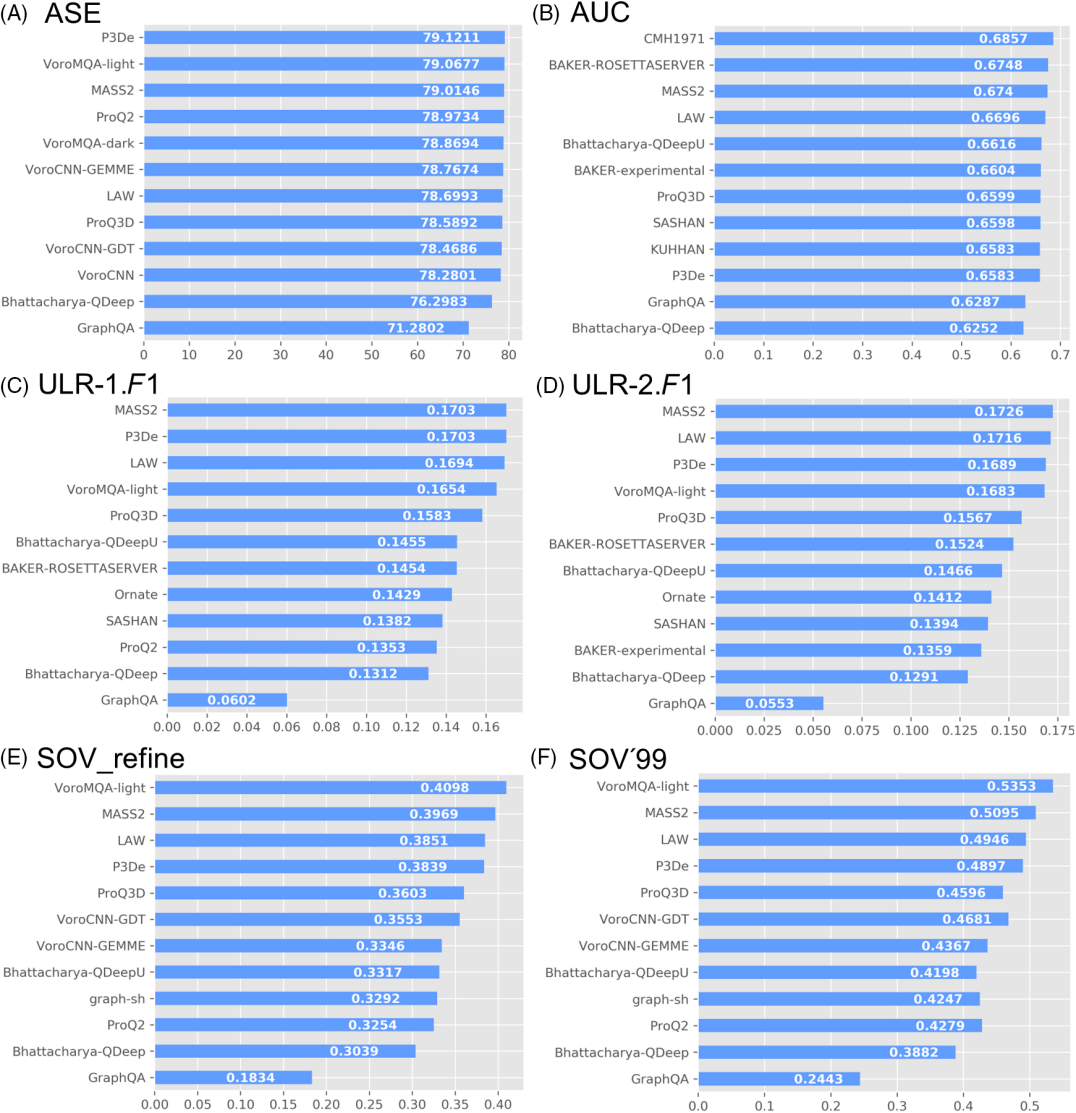
The ranking of selected CASP14 groups with the threshold of GDT_TS scores of TS models >40. (A–F) Shows the groups ranked by ASE, AUC, ULR‐1.*F*1, ULR‐2.*F*1, SOV_refine, and SOV'99, respectively

Figure 4 shows the ranking of CASP14 groups by ASE, AUC, ULR‐1.*F*1, ULR‐2.*F*1, SOV_refine, and SOV'99 for the threshold of GDT_TS scores of TS models >40. For each ranking, we show the top 10 performed methods and Bhattacharya‐QDeep and GraphQA for comparison. Tables S3–S7 shows the rankings of CASP14 groups for the other two thresholds of GDT_TS scores of the TS models (GDT_TS between 40 and 60, and any GDT_TS). In all rankings and three settings, both MASS2 and LAW rank in the top 10.

Particularly, in terms of the TS models with true GDT_TS scores >40, which were also what the CASP organizers used in their official evaluations, the rankings of MASS2 in ASE, AUC, ULR‐1.*F*1, ULR‐2.*F*1, and SOV_refine are third, third, first, first, and second, respectively. The corresponding rankings of LAW are seventh, fourth, third, second, and third, respectively. When we only evaluated on the average models (GDT_TS between 40 and 60), the MASS2 and LAW consistently rank in the top three. When all TS models of 90 EUs (CASP14 organizers used in total 90 EUs when evaluating CASP14 EMA predictions) were used for evaluation, MASS2 and LAW consistently rank in the top 4.

**FIGURE 5:**
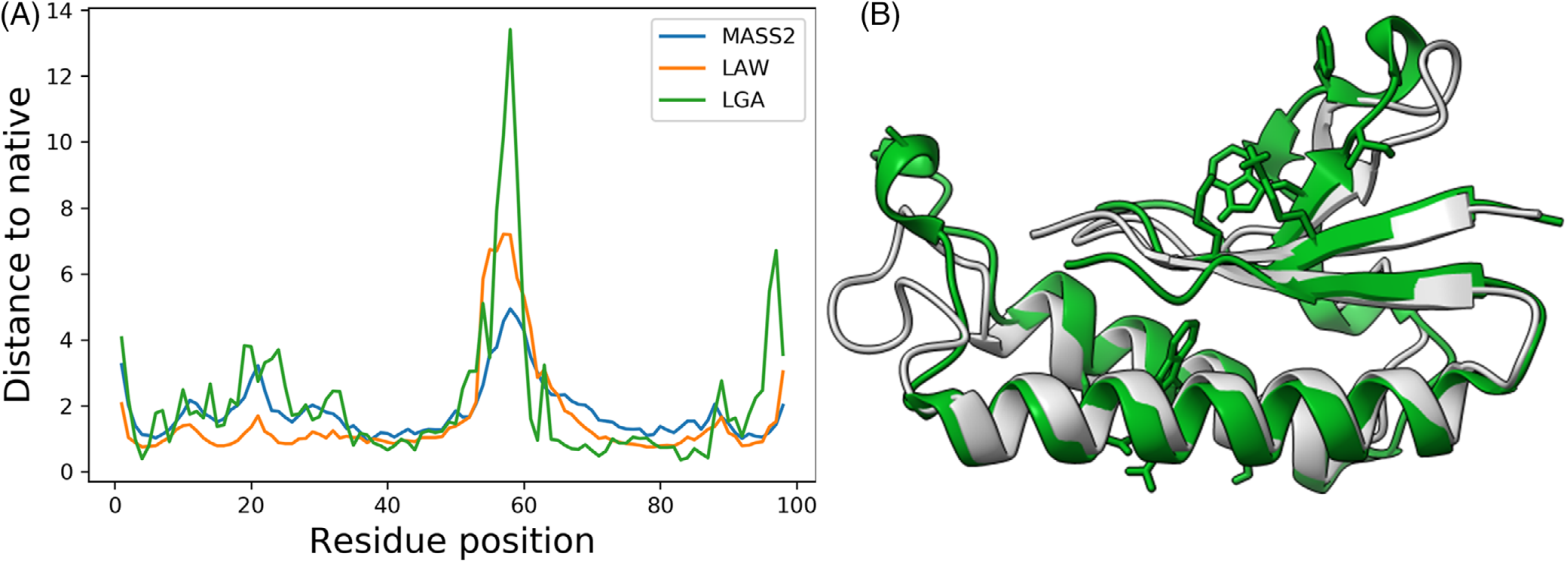
A local quality assessment example of MASS2 and LAW on a CASP14 model: T1065s2TS075_1‐D1. (A) Shows the native deviation (LGA) and the predicted deviations of MASS2 and LAW. (B) Shows the superposition of the native structure of target T1065s2 (green) and the model T1065s2TS075_1‐D1 (gray)

**FIGURE 6:**
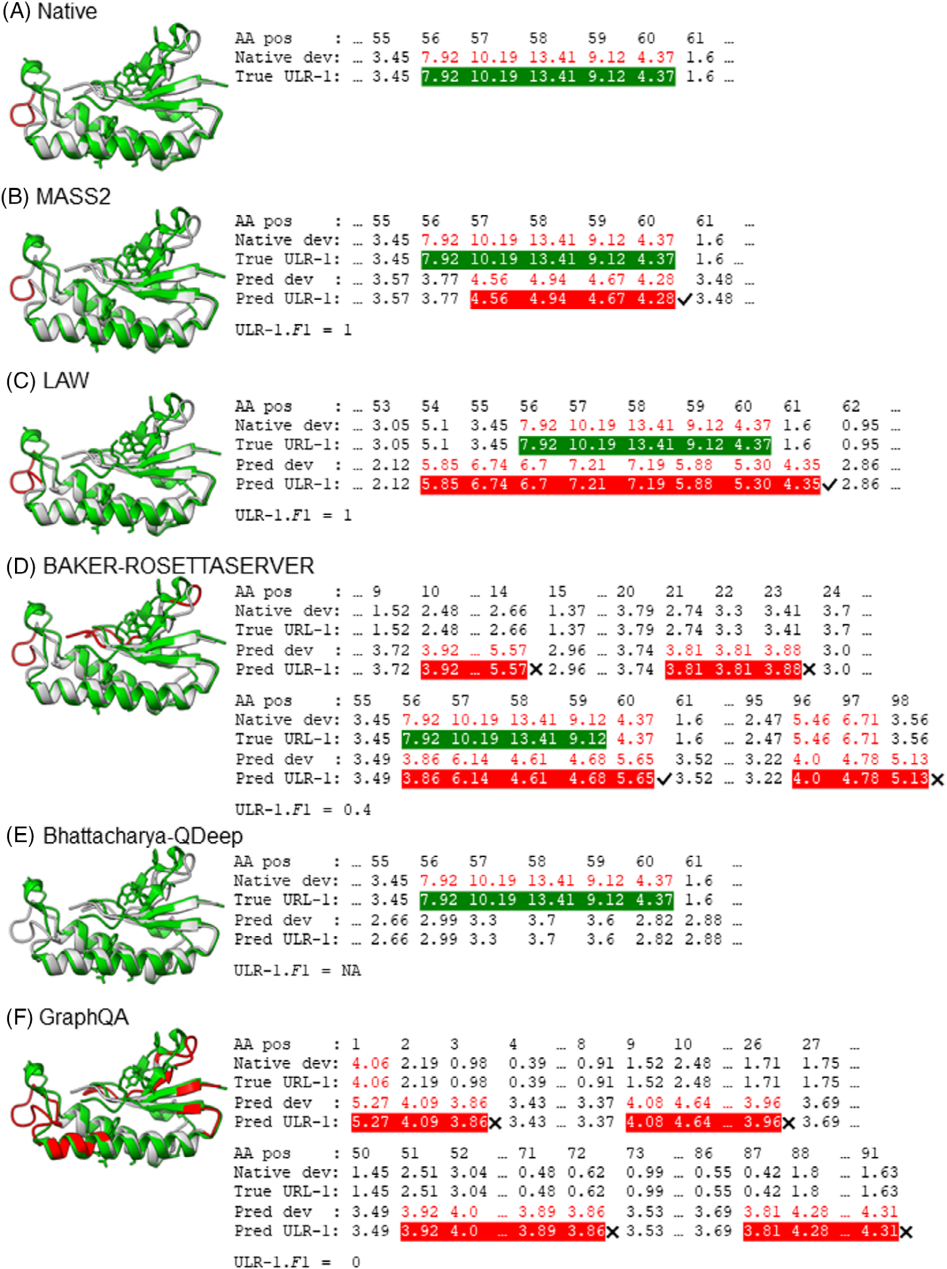
The ULR‐1 estimated by six CASP14 QA predictors for T1065s2TS075_1‐D1

We used two examples to show the performances of MASS2 and LAW on ASE and ULRs, respectively. Figure 5 shows the predicted distances $e_{i}$ of MASS2 and LAW and actual deviations $d_{i}$ for the target domain T1065s2TS075_1‐D1. Figure 5A shows the distance for each residue, and Figure 5B shows the superposition of the model and native structure. Figure 6 shows the ULR‐1s for T1065s2TS075_1‐D1 based on the estimated deviations generated by six CASP14 QA predictors, including our MASS2 and LAW.

## 5 DISCUSSION AND CONCLUSION

We designed two deep learning methods, named MASS2 and LAW, for estimating the residue‐specific qualities of individual protein models. MASS2 utilizes the residual networks, and LAW utilizes the graph networks. We benchmarked MASS2, LAW, and all other single‐model methods that participated in CASP14 on all TS models generated by server predictors. According to our evaluations, MASS2 is the best single‐model residue‐specific method in CASP14 based on the *Z*‐1 scores of ASE/100, AUC, and ULR‐1.*F*1, and the *Z*‐2 scores of ASE/100, AUC, ULR‐2.*F*1, and SOV_refine, whereas LAW is the second best single‐model residue‐specific method in CASP14 based on the *Z*‐1 scores of ASE/100, AUC, and ULR‐1.*F*1.

We also benchmarked the performance of MASS2, LAW, and the predicted deviations generated by AlphaFold2 on the TS models of AlphaFold2 in CASP14. Although LAW did not perform as well as MASS2 on the TS models of server predictors in CASP14, LAW outperformed MASS2 when evaluating the TS models of AlphaFold2 in CASP14. The QA performance of LAW on the models of AlphaFold2 is comparable to or even better than the QA performance of AlphaFold2. When users utilize our web servers, we may recommend using LAW for AlphaFold2 TS models and MASS for all other TS models.

We believe that the inclusion of spatial properties of protein models contributes to the promising performances of MASS2 and LAW. For MASS2, a unique type of feature that we have used is the concatenated features of the five spatially closest residues (sequential distances >6) of each target residue. These features were processed by three convolutional layers and then combined with other residue‐specific features by residual blocks. In this way, MASS2 is not only seeing residue‐specific features but also, to some degree, integrating the 3D structure of the TS model.

We had the same goal in mind when we designed LAW but on an even larger scale, that was, using a graph to represent the global 3D structure of the entire TS model, not only the five spatially closest residues as in MASS2. Since each edge in the graph is added to connect any two residues having a 3D Euclidean distance $\leq 8\;\overset{{}^{\circ}}{A}$, the graph topology, to some extent, is a representation of the key 3D structural properties of the model. The advantage of using a graph is that it does not include all of the 3D coordinates of the 3D structures, which decreases the complexity of modeling and reduces computational time.

## AUTHOR CONTRIBUTIONS

**Chenguang Zhao**: Conceptualization; Methodology; Software; Data curation; Validation; Formal analysis; Visualization; Writing – original draft; Writing – review and editing; Investigation. **Tong Liu:** Conceptualization; Methodology; Validation; Formal analysis; Writing – review and editing; Software; Investigation. **Zheng Wang**: Writing – review and editing; Conceptualization; Methodology; Formal analysis; Supervision; Funding acquisition; Project administration; Investigation.

## CONFLICT OF INTEREST

The authors declare that there is no conflict of interest.

## Supporting information


**Appendix S1** Supporting information.

**Data S1** Supporting information.

## Data Availability

The data that support the findings of this study are openly available in Dr. Zheng Wang's academic website at https://www.cs.miami.edu/home/zwang/software.html.
