# Supplementary material for: Predicting residue‐specific qualities of individual protein models using residual neural networks and graph neural networks
Source: Proteins. 2022 Jul 30;90(12):2091–102. doi: 10.1002/prot.26400 (PMC9796650; doi:10.1002/prot.26400)
Supplement: Supplementary file 1 — Appendix S1 Supporting information. [file PROT-90-2091-s001.pdf]

Predicting residue-specific qualities of individual protein models using residual neural networks and graph neural networks

Chenguang Zhao[1], Tong Liu[1], Zheng Wang[1,*]
Department of Computer Science, University of Miami
Zheng.Wang@miami.edu

Supplementary Figure S1. CASP14 groups ranked by the Z-2 scores. Z-2 score is the sum of averaged Z-scores of ASE/100, AUC, ULR-2.F1, and SOV_refine over all EUs.

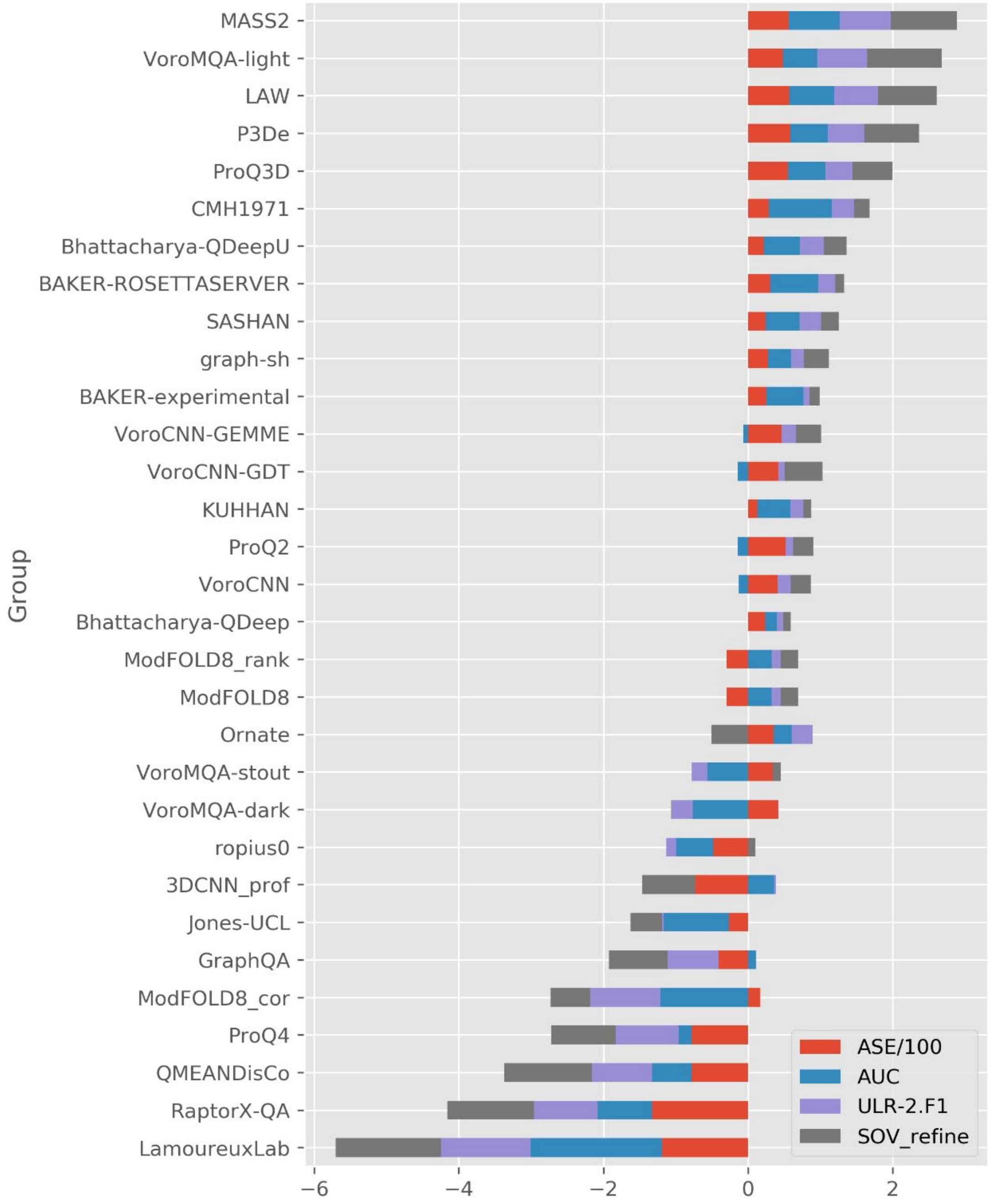

Supplementary Table S1. Datasets statistics of targets and models. We filtered out the models that have missing features in training and validation. The values before “/” are for MASS2, and the values after “/” are for LAW.

	GDT-TS	CASP round	Number of targets	Number of EUs	Number of models
Training	>40	7	57 / 55	82 / 55	24 909 / 20 245
	>40	8	120 / 111	159 / 138	46 818 / 38 084
	>40	9	111 / 121	133 / 102	42 040 / 35 076
	>40	10	88 / 79	108 / 94	25 664 / 20 963
	>40	11	79 / 71	102 / 84	14 940 / 12 086
Validation	>40	12	64 / 59	83 / 71	13 485 / 11 527
Benchmark	>40	14	63	90	10 306
	(40,60)	14	63	90	3803
	>0	14	63	90	13 500

Supplementary Table S2. Selected CASP14 groups ranked by the Z-2 scores. Z-2 score is the sum of averaged Z-scores of ASE/100, AUC, ULR-2.F1, and SOV_refine over all EUs.

Ranked by Z-2 scores	Groups	Z-2 scores
1	**MASS2**	2.887
2	VoroMQA-light	2.676
3	**LAW**	2.606
4	P3De	2.362
5	ProQ3D	1.995
6	CMH1971	1.678
7	Bhattacharya-QDeepU	1.359
8	BAKER-ROSETTASERVER	1.33
9	SASHAN	1.252
10	graph-sh	1.121
26	GraphQA	-1.814

Supplementary Table S3. Selected CASP14 groups ranked by ASE with two different thresholds of GDT-TS scores of TS models.

GDT-TS intervals	Rankings	Groups	Scores
(40,60)	1	**MASS2**	76.45905
	2	P3De	76.03877
	3	**LAW**	75.50498
	4	CMH1971	75.49532
	5	ProQ3D	75.42785
	6	VoroMQA-light	75.34338
	7	SASHAN	74.60809
	8	ProQ2	74.57945
	9	Ornate	74.30658
	10	VoroCNN	74.29103
	20	Bhattacharya-QDeep	72.47481
	21	GraphQA	70.73417
[0, 100]	1	ProQ3D	77.91314
	2	P3De	77.60372
	3	**MASS2**	76.98669
	4	**LAW**	76.96741
	5	ProQ2	76.57715
	6	Bhattacharya-QDeepU	76.42729
	7	BAKER-ROSETTASERVER	76.22091
	8	Bhattacharya-QDeep	76.1313
	9	VoroMQA-light	76.13112
	10	VoroCNN-GEMME	76.07783
	22	GraphQA	72.07661

Supplementary Table S4. Selected CASP14 groups ranked by AUC with two different thresholds of GDT-TS scores of TS models.

GDT-TS intervals	Rankings	Groups	Scores
(40,60)	1	CMH1971	0.65898
	2	**LAW**	0.65436
	3	**MASS2**	0.65204
	4	P3De	0.65093
	5	ProQ3D	0.64698
	6	VoroMQA-light	0.64276
	7	Bhattacharya-QDeepU	0.63873
	8	KUHHAN	0.63179
	9	SASHAN	0.62945
	10	ModFOLD8_rank	0.6251
	12	Bhattacharya-QDeep	0.62333
	22	GraphQA	0.57766
[0, 100]	1	CMH1971	0.672
	2	**MASS2**	0.668
	3	**LAW**	0.666
	4	BAKER-ROSETTASERVER	0.657
	5	Bhattacharya-QDeepU	0.656
	6	ProQ3D	0.652
	7	P3De	0.651
	8	KUHHAN	0.649
	9	SASHAN	0.648
	10	ModFOLD8	0.647
	16	GraphQA	0.621
	17	Bhattacharya-QDeep	0.62

Supplementary Table S5. Selected CASP14 groups ranked by ULR-1.F1 with two different thresholds of GDT-TS scores of TS models.

GDT-TS intervals	Rankings	Groups	Scores
(40,60)	1	VoroMQA-light	0.15209
	2	**MASS2**	0.14559
	3	**LAW**	0.14409
	4	CMH1971	0.12871
	5	P3De	0.12526
	6	Bhattacharya-QDeepU	0.12259
	7	ProQ3D	0.11986
	8	Ornate	0.11535
	9	SASHAN	0.11471
	10	KUHHAN	0.11281
	13	Bhattacharya-QDeep	0.10348
	27	GraphQA	0.0391
[0, 100]	1	P3De	0.16514
	2	**LAW**	0.16248
	3	**MASS2**	0.16175
	4	ProQ3D	0.1555
	5	VoroMQA-light	0.1531
	6	Bhattacharya-QDeepU	0.14127
	7	BAKER-ROSETTASERVER	0.13653
	8	Ornate	0.13411
	9	SASHAN	0.13144
	10	ModFOLD8_rank	0.12917
	13	Bhattacharya-QDeep	0.12848
	26	GraphQA	0.05715

Supplementary Table S6. Selected CASP14 groups ranked by ULR-2.F1 with two different thresholds of GDT-TS scores of TS models.

GDT-TS intervals	Rankings	Groups	Scores
(40,60)	1	VoroMQA-light	0.15479
	2	**MASS2**	0.14713
	3	**LAW**	0.14611
	4	CMH1971	0.13518
	5	P3De	0.12534
	6	Bhattacharya-QDeepU	0.12296
	7	Ornate	0.12046
	8	ProQ3D	0.11932
	9	SASHAN	0.11805
	10	ModFOLD8	0.11785
	13	Bhattacharya-QDeep	0.09851
	27	GraphQA	0.03784
[0, 100]	1	**LAW**	0.16453
	2	**MASS2**	0.16447
	3	P3De	0.16442
	4	VoroMQA-light	0.15625
	5	ProQ3D	0.15371
	6	Bhattacharya-QDeepU	0.14331
	7	BAKER-ROSETTASERVER	0.14268
	8	ModFOLD8_rank	0.13489
	9	ModFOLD8	0.13486
	10	SASHAN	0.13363
	13	Bhattacharya-QDeep	0.1264
	27	GraphQA	0.0529

Supplementary Table S7. Selected CASP14 groups ranked by SOV_refine with two different thresholds of GDT-TS scores of TS models.

GDT-TS intervals	SOV_refine rankings	Groups	SOV_refine	SOV'99	SOV'99 rankings
(40,60)	1	VoroMQA-light	0.38135	0.4963	1
	2	**MASS2**	0.37051	0.47556	2
	3	**LAW**	0.35489	0.45717	3
	4	P3De	0.33267	0.4267	4
	5	CMH1971	0.31835	0.41134	5
	6	ProQ3D	0.31599	0.40663	6
	7	SASHAN	0.29925	0.38819	7
	8	Bhattacharya-QDeepU	0.29776	0.37812	10
	9	KUHHAN	0.29304	0.37993	9
	10	ModFOLD8	0.28931	0.36889	11
	15	Bhattacharya-QDeep	0.26713	0.34057	17
	27	GraphQA	0.16561	0.22147	27
[0, 100]	1	VoroMQA-light	0.38695	0.50807	1
	2	P3De	0.3862	0.49462	2
	3	**MASS2**	0.38151	0.4915	3
	4	**LAW**	0.36892	0.47461	4
	5	ProQ3D	0.36102	0.46268	5
	6	ModFOLD8_rank	0.3463	0.44	7
	7	ModFOLD8	0.34629	0.44	8
	8	VoroCNN-GDT	0.34411	0.45511	6
	9	Bhattacharya-QDeepU	0.34254	0.43635	9
	10	BAKER-ROSETTASERVER	0.31829	0.40767	13
	16	Bhattacharya-QDeep	0.31208	0.40057	18
	24	GraphQA	0.21253	0.28675	24
